# Human Sensorimotor Communication: A Theory of Signaling in Online Social Interactions

**DOI:** 10.1371/journal.pone.0079876

**Published:** 2013-11-20

**Authors:** Giovanni Pezzulo, Francesco Donnarumma, Haris Dindo

**Affiliations:** 1 Institute of Cognitive Sciences and Technologies, National Research Council, Rome, Italy; 2 Computer Science Engineering, University of Palermo, Palermo, Italy; Brain and Spine Institute (ICM), France

## Abstract

Although the importance of communication is recognized in several disciplines, it is rarely studied in the context of online social interactions and joint actions. During online joint actions, language and gesture are often insufficient and humans typically use non-verbal, sensorimotor forms of communication to send coordination signals. For example, when playing volleyball, an athlete can exaggerate her movements to signal her intentions to her teammates (say, a pass to the right) or to feint an adversary. Similarly, a person who is transporting a table together with a co-actor can push the table in a certain direction to signal where and when he intends to place it. Other examples of “signaling” are over-articulating in noisy environments and over-emphasizing vowels in child-directed speech. In all these examples, humans intentionally modify their action kinematics to make their goals easier to disambiguate. At the moment no formal theory exists of these forms of sensorimotor communication and signaling. We present one such theory that describes signaling as a combination of a pragmatic and a communicative action, and explains how it simplifies coordination in online social interactions. We cast signaling within a “joint action optimization” framework in which co-actors optimize the success of their interaction and joint goals rather than only their part of the joint action. The decision of whether and how much to signal requires solving a trade-off between the costs of modifying one’s behavior and the benefits in terms of interaction success. Signaling is thus an intentional strategy that supports social interactions; it acts in concert with automatic mechanisms of resonance, prediction, and imitation, especially when the context makes actions and intentions ambiguous and difficult to read. Our theory suggests that communication dynamics should be studied within theories of coordination and interaction rather than only in terms of the maximization of information transmission.

## Introduction

The study of human communication is central in several disciplines, including linguistics, cognitive science, neuroscience, anthropology, biology, and philosophy [1]–[5]. Most studies have focused on linguistic communication or non-linguistic forms such as gesture, deictics (e.g., pointing, using turn signals when driving, or looking at objects to help another’s referential processes), facial expressions, body language and posture.

Less attention has been devoted to *human sensorimotor communication* during online social interactions and joint actions, such as for instance when two persons play volleyball or jointly lift a table. Unlike conversation, these joint actions are not exclusively communicative but have non-communicative, pragmatic goals (winning a match or transporting the table somewhere). Still, as recognized by early scholars [4], [6]–[8] and also more recently [9], joint actions offer a vantage point from which to study communication and its origins.

In online sensorimotor interactions and joint actions, communication is not only linguistic. Several studies have focused on other forms of communication, which include deictics, gesturing, and facial expressions, see [10] for a recent review. A common feature of all these forms of communication is that the “channel” used for communication (say, language or gaze) is different from the channel used for action (say, the movements required to pass the ball in a volleyball game or to lift a table). In this article we focus instead in a less-studied form of communication that uses the same channel as the to-be-executed action. Consider the case of a volleyball player exaggerating her movements to help her teammates discriminating between a pass to the right or left. In this case, the same channel (i.e. hand and body movements) is used for both reaching/lifting and sending coordination signals to the co-actor.

How can we distinguish when a given action (say, making a pass) is used for its pragmatic goal (e.g., passing the ball to the teammate) or for *signaling* something to a co-actor (e.g., letting her infer the direction of the pass)? Evidence accumulates that distinguishing features can be found at the kinematic level. For example, it has been reported that in social contexts actions have subtle still significant kinematic peculiarities: the deceleration phase was slower when a “giving” action was directed to another individual that in an individualistic set-up (placing an object) [11]. In turn, these subtle changes in the action kinematics are highly informative of the performer’s goals and might help inferring their distal intentions; thus, they have communicative and not only pragmatic effects [12]–[14]. Other studies have reported that co-actors engaged in joint actions modify the kinematics of their actions, and in particular make their behavior more predictable and discriminable [15]–[18]. This can be done by minimizing its variance [19] or selecting trajectories that permit a faster disambiguation of the action from the alternatives [16], [17]. In a previous study we found that in joint actions with asymmetric knowledge (e.g., only one of the co-actors knew the joint goal) the knowledgeable subject modified her actions, and stopped doing so when the co-actor’s uncertainty lowered, showing that the signaling mechanisms can adapt flexibly to task demands [20]. Note that signaling is not limited to one modality but can exploit multiple communication channels such as visuomotor, auditory, and haptic coupling [21]. Furthermore, signaling can be used at a larger scale than in the previous examples; for instance in the coordination of an entire orchestra, and be responsible for the quality of the artistic results [22].

Sensorimotor strategies of communication can also work synergistically with other forms of communication (e.g., linguistic). It has been consistently reported that child-directed speech (a “motherese”) has certain characteristics that facilitate recognition and perceptual processing, such as for instance an exaggeration of the vowels [23]. Mothers’ infant-directed action (“motionese”) has similar characteristics of the motherese, including the “exaggerations” and the choice of actions that have low perceptual ambiguity [24], [25]. Exaggeration is not the only strategy; speaking slower achieves the same goal. Besides teaching, signaling helps conveying communicative intentions when the context makes them ambiguous. For example, interlocutors often over-articulate and modulate their choice of words to help the receiver’s perceptual processing in noisy environments (the so-called *Lombard effect*). Studies of sign language reveal *dissimilation* effects, too: performers chose movement parameters that make two successive actions more easily discriminable for the perceiver [26]. Exaggeration of movements can be used to convey misleading communicative messages, too, such as when feinting in soccer [27], [28].

Despite these empirical demonstrations, the nature and functioning of human sensorimotor communication is largely unknown. There is currently no normative framework or analytic description of human signaling and sensorimotor communication that provides them with a theoretical ground. Besides its importance per se, the study of sensorimotor communication provides an excellent opportunity to understand the adaptive and evolutionary value of communication in terms of coordination and interaction success. Indeed, joint action scenarios are interesting because they are not essentially communicative but have first and foremost pragmatic goals. This makes it possible to study the trade-offs between the costs and benefits of communication in terms of interaction success, not (only) of communication success, as it is instead common in theories focusing on maximization of information transmission.

### Overview of the Proposed Theory

In this article we present a formal theory of signaling in online sensorimotor interactions. Signaling refers to the intentional modification of one’s own behavior to convey information to another person, typically a co-actor. In addition to a pragmatic goal (e.g., performing the joint task), the signaler generally has the communicative goal of changing the co-actor’s belief (e.g., facilitate his understanding of the signaler’s goals). In some cases, but not always, the signaler wants also the co-actor to be aware that she is intentionally communicating or cooperating. Note that in competitive scenarios the signal can also be misleading (e.g., a feint in soccer [28]). Note that our use of the term “signaling” is different from the use in animal communication, because it is intentional and tailored to the addressee and his uncertainty; rather, the jump of a gazelle signals its strength but is less flexible and can be executed irrespective of the presence of a mate or a lion [29], [30].

The proposed theory starts from the premise that in joint action contexts co-actors optimize the success of joint goals (e.g., lifting a table together) rather than their individual ones (e.g., lifting my part of the table), and take into consideration the joint outcome (e.g., ensure that the table surface is horizontal) and when necessary also the parameters of the co-actor (trajectories, sensory feedback, uncertainty, task knowledge) rather than only their own. Signaling is part of this *joint action optimization*. To optimize the joint goal, the actor can choose to pay a cost in terms of her individual performance. For example, to make her action more predictable and discriminable, she can chose an exaggerated trajectory requiring higher muscular effort or a biomechanically awkward posture.

We formalize signaling in terms of parametrizable deviations from the action’s optimal trajectory so that the signaling action retains its pragmatic goal (e.g., grasping an object) but the changes in the kinematic parameters are informative of the performer’s action choice. To choose whether or not to signal, and how much to signal, subjects perform a cost-benefit analysis (considering, for instance, the uncertainty of the co-actor, the accuracy of the end state if a signaling action is selected, the usefulness of signaling by varying one or more parameters). As it requires a cost-benefit analysis, we consider signaling as an intentional form of sensorimotor communication, not a byproduct of interaction dynamics.

Our theory makes four assumptions. First, the computational objective of signaling is permitting the co-actor to better discriminate the signaler’s action goal (and/or distal intention) against the alternatives (i.e., raising the co-actor’s 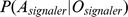, where 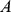 is the action goal of the signaler and 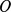 is the observed movement of the signaler). As we will discuss, because we assume that perceivers use predictive strategies to recognize the performer’s action goal, the signaling action has to improve the perceiver’s predictions. Second, the decision of wether or not and how much to signal requires solving a trade-off between the immediate costs of signaling (e.g., the biomechanical costs linked to the execution of an awkward trajectory) and its benefits in terms of interaction success. Third, signaling actions consist in the *dissimilation* (or differentiation) of one’s own action parameters (e.g., trajectory, speed, or hand size) from those of the alternative actions that are most likely given the context and the perceiver’s prior information. Fourth, our parametrization permits to select different “degrees” of signaling actions, and to consider their costs (roughly, the bigger the deviation from the optimal trajectories, the higher the cost) in addition to their benefits (in terms of how much signaling facilitates the discrimination of different actions by the perceiver).

## Methods

### Computational Framework

The theory starts from the premise that a performer agent wants to influence (typically facilitate) the action recognition process of a perceiver agent. Consider the case of a volleyball player who can execute one of two possible action alternatives: passing the ball to the left or right. A teammate sees the scene but is uncertain relative to the performer’s action: action 1 (say, pass to the left) or action 2 (say, pass to the right). The teammate can estimate the most likely action of the performer based on multiple sources of information, such as the performer’s arm trajectory, body posture, hand movements, gaze direction, and various kinds of prior information (e.g. the performer’s preference and skills). The essence of our signaling theory is that the performer can *purposively* modify one or more of said variables for communicative purposes; for example, to help the teammate recognize and predict the performed action.

Let’s describe more formally the computational problems that performer and perceiver must solve. Each goal-directed action can be performed in a number of different ways (e.g. a pass to the left can be achieved in several ways). An authoritative view in computational motor control is that each particular instantiation of an action can be associated to an internal model 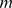 [31], [32]. In our approach, the internal model maps to a probabilistic trajectory; each trajectory is associated to a goal-directed action (e.g., reaching an object to the left or right). From the computational point of view, each model (and thus a particular action choice) can be represented as a stochastic process modeling the temporal evolution of the system’s state. Without loss of generality, we can represent the evolution of a model as 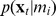, where 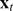 is the state of the system at time t (e.g. the value of the performer’s kinematic parameters at time 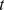). By 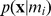 instead, we denote the entire sequence of states of the system during the action associated to the model 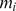.

During an interaction, the perceiver’s goal is to infer which model 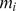 has generated the observed data by inverting the generative model above (this implies that interacting agents share similar models, and are thus able to perform similar goal-oriented actions, see [33]). In other words, the perceiver’s goal is to infer:
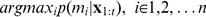
(1)where 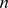 is the number of available models (generally, the state 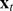 of the system is not directly observable. Rather, a set of variables 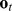 is observed and the distribution linking those variables to the state, 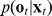, is supposed to be known). For a computationally efficient solution to this problem please consult [33].

The performer’s goal, on the other hand, is to facilitate such an inferential process. Here we argue that this result can be achieved by actively modifying the parameters of the currently executed model, and thus the resulting trajectory 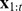, in order to minimize the probability of a misclassification in Equation 1. We call this process *signaling*.

### Signaling Distribution

Signaling can be defined as the process of altering one’s own behavior to facilitate its recognition by other persons. In our framework, a signaling agent tries to “dissimilate” her trajectory from the alternative ones that are possible (or likely) given the context [26], where *dissimilation* is defined as the amplification of the differences between the selected trajectory and the alternative one(s). At the same time, however, the action choice has to be a valid one to preserve its pragmatic effect (i.e., not to fail the action) and make the recognition by the perceiver possible. Thus, samples from the signaling distribution for a given model 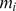 at time 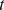 shall be representative of the “original” distribution 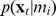 while having a low probability of being sampled from other competing models, 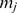, in a given context.

In line with the requirements above, samples from the signaling distribution can be obtained via a modified *rejection sampling* technique [34]. Let 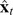 be a sample from the “ideal” distribution 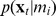. Given 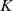 random values, 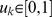, sampled from the uniform distribution over 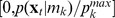, we decide to *accept* the sample 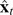 if the following holds:
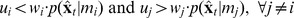
(2)where 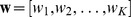 is a vector of weights which modulate the contribution of individual models to the the signaling distribution.

In the case of continuous distributions 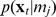 the procedure above leads to the formal definition of the *signaling* distribution:
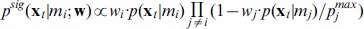
(3)where 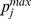 is the maximum value for the distribution 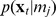. By means of 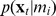 it is possible to compute the entire sequence of states of the system during the signaling of the action associated to the model 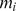 which we denote with 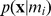.

### To Signal or not to Signal (and How Much to Signal)

Intuitively, the signaling distribution for the model 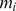 should be as close as possible to the original distribution 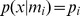, while having at the same time a high discriminative power as to facilitate action recognition. This can be formally represented as an optimization problem where the goal is to find the components of the weight vector 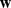 of the signaling distribution which minimizes the following:
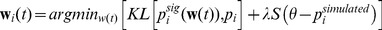
(4)where:

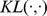
 is the Kullback-Leibler divergence between the signaling distribution with the set of weights 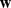 and the original one (i.e., the one with no signaling);

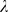
 is the amount of signaling in the given action;

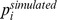
 is an estimation of the perceiver’s posterior probability of correctly recognizing the model 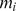 under the assumption that performer and perceiver share the same set of internal models;

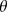
 is the experimentally fixed threshold that the perceiver uses during model recognition (in our experiments, 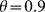);

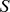
 is the logistic function.

The minimization of Eq. 4 considers jointly three factors. The KL term considers the cost of signaling, where cost can be associated to biomechanical factors, effort, and other forms of costs (e.g., cognitive costs associated to planning and executing non-familiar or non-habitual movements). The 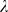 term permits to modulate the amount of signaling, where 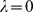 means no signaling. Note that our formulation permits to modulate the amount of signaling during the task, for example signaling only during the first part of the action or stopping signaling when the perceiver’s feedback indicates that it is not necessary. The 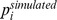 term describes a simulation of the perceiver’s action recognition process (for mathematical details of how to efficiently compute this probability please refer to [20], [33]. It permits estimating the potential benefit that the perceiver will have from signaling actions of having different 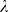, where the benefit consists in better and faster recognition of the executed action. All these factors are jointly considered in the cost-benefit analysis that selects whether or not, and how much to signal.

### Example of Signaling Distribution

As an example of signaling distribution, consider a system governed by two independent Gaussian processes corresponding to models 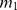 and 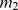, respectively. Assume that the system commits to the model 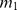, the corresponding signaling distribution is given by:
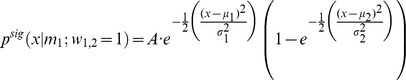
with



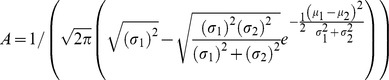



For the sake of simplicity we assume that the contribution of each internal model to the state can be expressed by a Gaussian distribution with mean 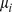 and a standard deviation 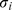:
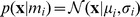



However, the theory is independent from the particular density function chosen.

## Results

To test our model, we performed three experiments. The first experiment uses a synthetic dataset to tests if, when the performer uses the signaling strategy elucidated earlier, the perceiver can better discriminate the right action from an alternative one. The second experiment consists in using our proposed approach to model human data on a joint action task that requires signaling. The third experiment is similar to the first experiment but considers the case of three actions to be disambiguated.

### Experiment 1: The Effects and Benefits of Signaling

**Figure 1:**
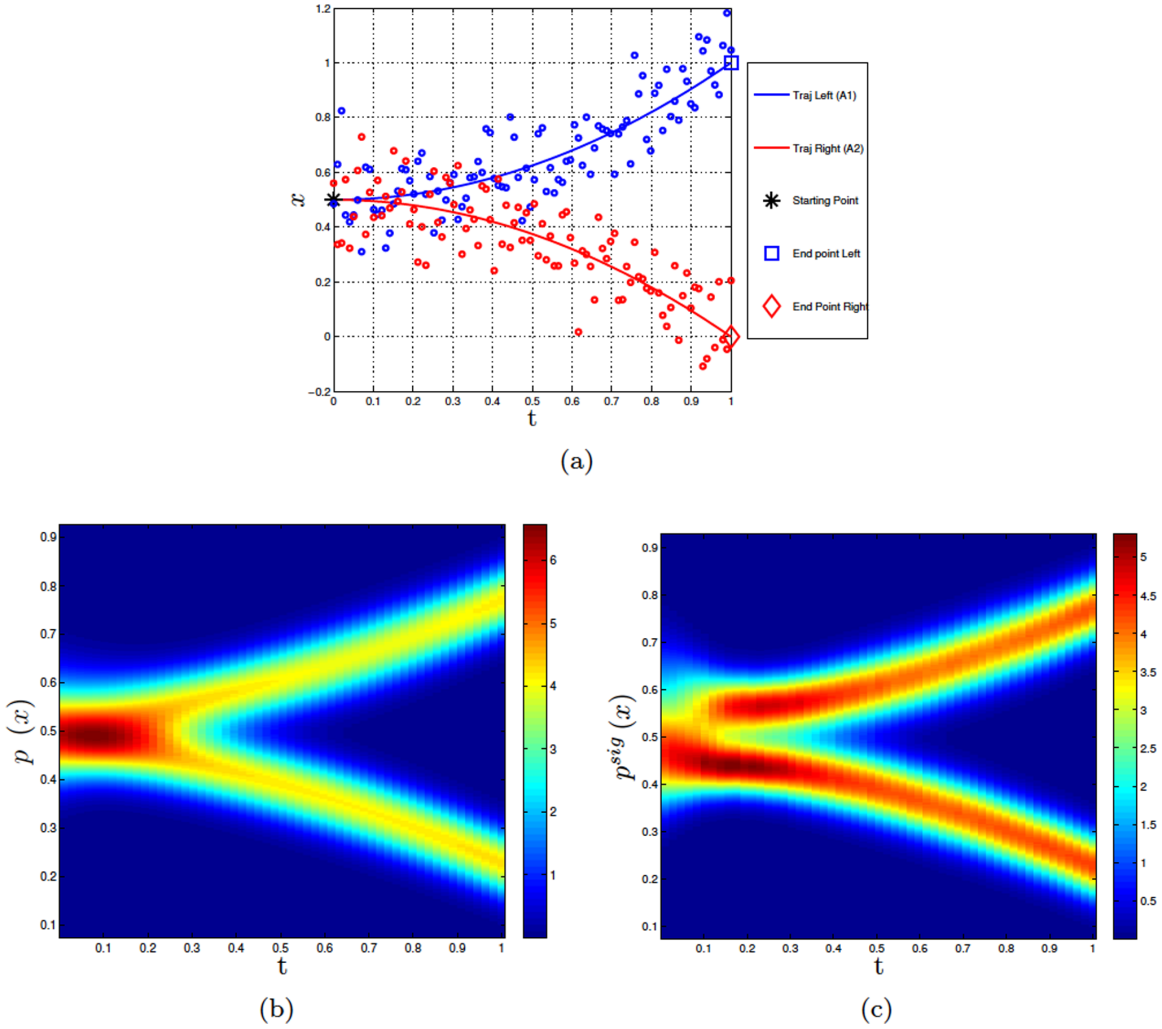
Experiment 1. Panel a shows the dataset composed of synthetic data points for two goal-directed actions: 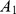 (e.g. reach an object to the left) and 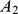 (e.g. reach an object to the right). The lines show the trajectories from which the noisy data (shown as circles) were sampled. Synthetic points were generated by adding Gaussian noise to each trajectory with a signal-to-noise ratio of 18dB. The trajectories were generated with a strong superposition over the 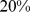 of their time length, in order to simulate movements not simply distinguishable. Panel b shows the probability density of the superposition of the two original models internal models 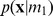 and 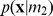 learned on the dataset of Panel a. The red color represents the highest probability density values, and the blue color the lowest. Panel c shows the corresponding trajectories of the signaling distributions 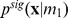 and 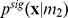.

To test the efficacy of the proposed signaling model in terms of improved action recognition, we designed an experiment using a synthetic dataset with two trajectories corresponding to two goal-directed actions: 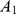 (e.g. reach an object to the left) and 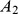 (e.g. reach an object to the right). These can be considered as idealization of passing actions of the volleyball player (passing the ball to the left or right). Figure 1a shows the synthetic data points for the two trajectories corresponding to 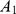 and 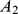. Full lines show the means of the trajectories, while the points represent the noisy data forming the dataset.

Figure 1b shows the trajectories of the two probabilistic internal models 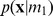 and 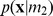 (where 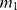 is the internal model for 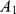 and 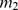 is the internal model for 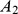) as learned by using the Echo State Gaussian Process algorithm [35] on 100 time series per action in a supervised learning scheme. These are considered to be the “original” internal models and trajectories (i.e., without signaling) in our experiment. The superposition of the two distributions in the figure represents the perceiver’s probability of observing the two movements when no hypothesis is done on which is the action actually being performed. Figure 1c shows the internal models for the same two actions (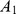 and 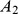) when the *signaling distribution* is used instead. By comparing figure 1b (without signaling) and figure 1c (with signaling) it can be noted that sampling from the signaling distribution leads to a characteristic dissimilation effect at the beginning of the action.

**Figure 2:**
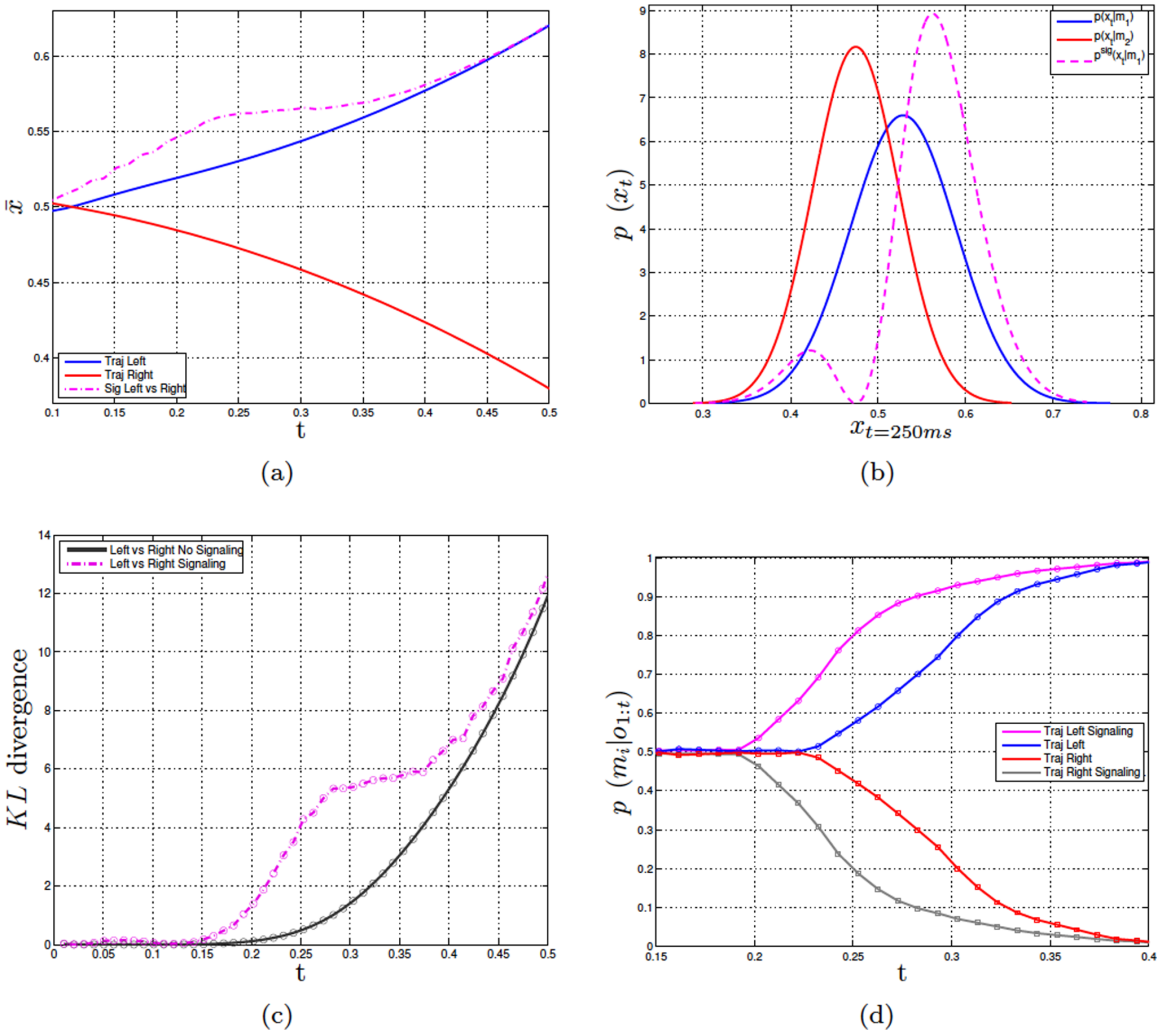
Further analyses of Experiment 1. Panel a shows the means of the original distributions for 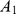 and 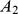 in blue and red, respectively. In magenta the means of the signaling distribution of 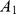 (i.e., for a leftward movement) is shown. Panel b shows an example of signaling distribution at a given time step rather than the whole trajectory. Sample distributions 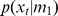 and 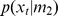 are taken at time 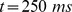 of the dynamic Gaussian Process of the two primitives. The parameters of the Gaussian distributions at time 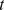 are 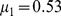, 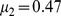, 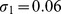 and 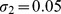. The resulting distribution 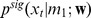 is computed from Eq. 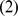. The weights coefficients are set as 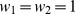. This means that the two distributions are equally weighted in the computing of the signaling distribution. Panel c shows the KL divergence between two actions: 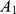 vs. 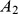. Panel d shows the perceiver’s probability of recognizing the right action (i.e., the probability 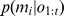 of perceiving 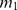 given the observations 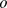 until time 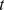) when the performer uses the original 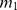 distribution (blue = left, red = right) and the signaling distribution (magenta = left, black = right).

Figure 2a and figure 2b permits to better appreciate this dissimilation effect. Figure 2a shows the difference between the trajectories for the action 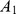 when the original distribution (blue) or the signaling distribution (magenta) are used. Figure 2b shows the same difference between the distributions, but at a particular time step 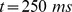. As evident from the figures, the differences between the original and the signaling distributions are not the same throughout the action but are more prominent at the beginning, where the trajectories of 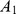 and 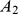 overlap. To assess this difference quantitatively, we calculated the Kullback-Leibler (KL) divergence between the correct and incorrect actions (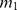 vs. 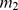) in the original and signaling distributions, see Figure 2c. Our results indicate that, after a short initial phase where ambiguity cannot be minimized, the signaling distribution quickly diverges from the original one; at the end of the action the signaling trajectory becomes close again to the original trajectory.

We argued that the effect of signaling and dissimilation is minimizing the probability of misclassification of a perceiver agent. To assess this hypothesis, we compared the performance of a (synthetic) perceiver agent asked to recognize a performed action when its trajectory is sampled from (i) the original distribution and (ii) a *signaling distribution*. The synthetic perceiver agent is modeled using a Bayesian action recognition method that integrates over time (noisy) trajectory estimates, see [33] for details.

Figure 2d shows the results of the comparison and the probability recognizing the two actions 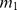 and 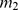 using the original distribution (blue and red) and the signaling distribution (magenta and black). The results indicate that the observer agent is able to recognize the correct action faster when a signaling distribution is used. Taken together, the results of this synthetic experiment show that using signaling strategies permitting dissimilating the two actions in the parts where their trajectories are more ambiguous; in turn, signaling permits perceivers to recognize a performed action faster and more accurately.

### Experiment 2. Comparison with Human Data

**Figure 3:**
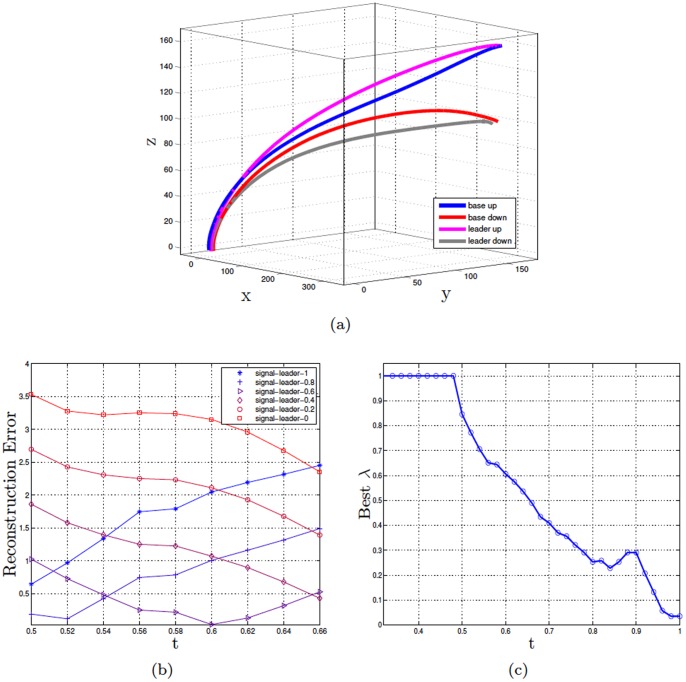
Experiment 2. Panel a shows the average trajectories (Leader vs. baseline condition) to reach the top and bottom of the bottle in the human study reported in [17]. Panel b shows the reconstruction error (sum of squares error between the human data and the modeled trajectories) for the trajectories of the Leader, using the signaling distribution and various values of 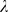. The Figure shows a time window in the data, not the whole trajectory. The specific window is between 0.5 and 0.66 (trajectory length is normalized to 1). Panel c shows a 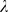-*profile*: the values of optimized 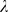 for each time step 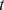 on the sequences of the Leaders’ reaching up movements. It measures the amount of signaling during the reaching up action.

The objective of experiment 2 is is assessing if our method can be used to model human signaling data. A recent experiment [17] investigated signaling in a joint task consisting in grasping jointly and synchronously a bottle-like object in one of two possible points: up (top of the bottle) or down (bottom of the bottle). In one condition, only one of the co-actors, called the *Leader*, knows the task to be jointly performed (i.e., up-reach or down-reach). Results show that in this case the *Leader* modifies her trajectory to signal her co-actor, called the *Follower*, the task to be performed. Specifically, if the *Leader* is required to do an up-reaching, she significantly raises the trajectory; if she is required to do a down-reaching, she significantly lowers the trajectory. Figure 3a shows a plot of the trajectories obtained in the human study and permits appreciating the significant differences between the signaling trajectories and the baseline trajectories (i.e., when no signaling is required).

We used the proposed signaling method to model the human data reported in [17]. We firstly acquired the internal models corresponding to the baseline trajectories (i.e., without signaling) for *reaching up* and *reaching down*. This was done by using the baseline human data in a supervised learning scheme, adopting the Echo State Gaussian Process algorithm [35].

Then, we calculated various *signaling distributions* using different values of 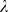, and compared the results to the Leader’s movements in the human study. Figure 3b shows that without signaling (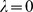) the reconstruction error is higher. The reconstruction error drops close to zero with various values of 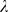, and the best model of the data is one in which 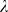 changes dynamically over time; see the dynamics of the 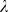-*profile* in figure 3c. This result suggests that our signaling method can successfully model the unfolding of the Leader’s actions over time. Furthermore, it indicates that the Leader might modify dynamically her signaling strategies within trials. One possibility is that the Leader does so depending on the Follower’s feedback. For example, a Follower could start the trial with a high level of uncertainty, prompting the Leader’s signaling. During the trial, the Follower could understand the correct action and move to the target with increased confidence; the Leader can use this feedback information to infer that the Follower has no uncertainty and stop signaling. Note that biomechanical constraints and other costs plausibly exert an influence on the dynamics of 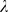. Indeed, the costs of deviating from the original trajectory are not constant within trials; in particular, they could increase at the end of the trajectory when it is important not to miss the pragmatic goal (e.g., grasping the bottle correctly). Future studies should address the costs and dynamics of signaling within and across trials.

### Experiment 3: The Case of Three or More Actions

Up to now we have considered the case of two actions 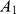 and 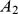. However, in realistic scenarios each context is linked to multiple possible actions. In such cases signaling strategies need to be different depending on what are the (most likely) action alternatives, say 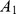 vs. 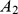 or 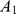 vs. 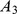 (or even 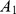 vs. both 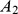 and 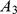). The aim of Experiment 3 is studying if and how signaling strategies change depending on the action alternatives.

**Figure 4:**
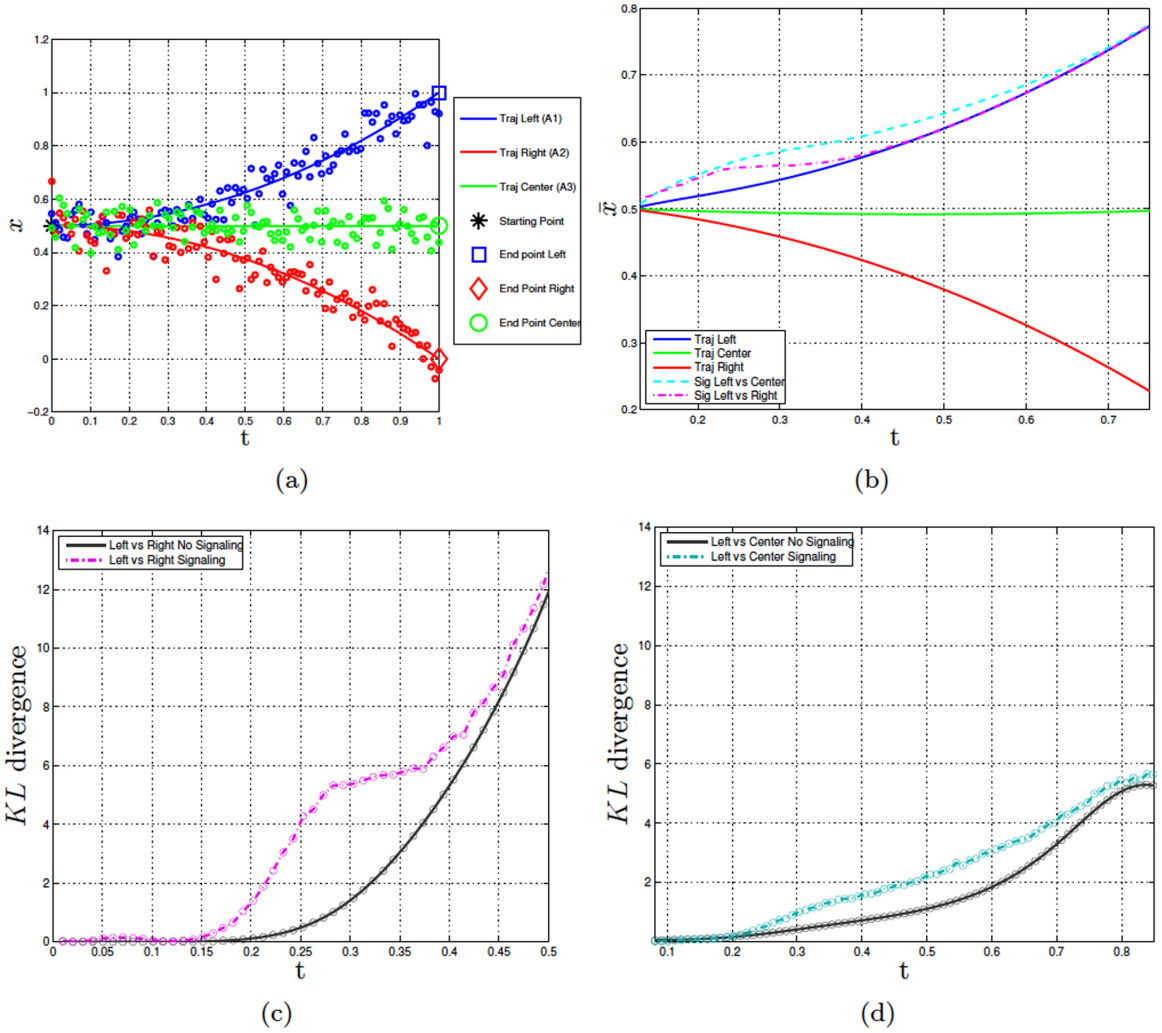
Experiment 3. Panel a shows the dataset of the synthetic data of the trajectories for actions 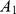 (left), 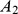 (right), and 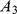 (center). The lines show the sample trajectories from which the noisy data (shown as circles) were sampled. Panel b shows the means of the original distribution of 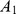 (blue), the means of the original distribution of 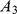 (green), the means of the original distribution of 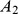 (red), the means of the signaling distribution 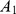 vs. 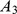 (turquoise), the means of the signaling distribution 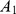 vs. 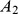 (magenta). Panels c and d compare the KL divergence of the original and signaling distributions in the case of 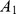 vs. 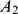 and 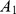 vs. 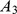, respectively.

Figure 4 illustrates this situation in the case of three actions (say, three goal-directed actions to the left, center, and right). In particular, Figure 4a shows the trajectories associated with the three actions, generated using a synthetic dataset that uses the method elucidated earlier but includes three rather than two actions. Figure 4b shows the means of the distributions for 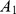, 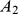, and 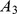 (in blue, red and green, respectively). It shows that trajectories using the sampling distribution are different if the same action 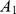 has to be dissimilated from 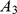 (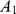 vs. 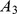 shown in turquoise) or from 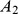 (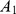 vs. 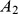 shown in magenta). In particular, a larger and longer deviation from the original trajectory is needed to dissimilate 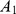 from 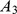 than 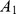 from 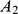. This difference can be appreciated by comparing figure 4c and figure 4d that compare the KL divergence of the original and signaling distributions in the case of 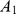 vs. 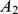 and 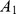 vs. 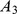, respectively.

These results show that signaling strategies are flexible and tailored to the action alternatives. The success of a signaling action depends on the action alternatives that are considered. Let’s imagine that a perceiver action is uncertain between A1 and A3. If the performer uses a signaling action that disambiguates between A1 and A2 rather than A1 and A3, he would stop signaling too early and this would make the perceiver’s task harder. We hypothesize that the performer modifies its signaling strategies using an estimation of the perceiver’s belief of the possible alternatives and the associated uncertainty. This novel empirical prediction of our model remains to be studied empirically. However, ongoing joint action experiments with several action alternatives show that the leader’s movement amplitude contains information about the target location (which is unknown to the co-actor) and the amplitude increases when the target-to-target distance increases (Cordula Vesper, personal communication).

## Discussion

Although the importance of communication is recognized in several disciplines, it is rarely studied in the context of on-line social interactions and joint actions. We offered a normative framework in which a performer agent can decide to pay a cost in order to send coordination signals and help the co-actor’s recognition process. In this framework, signaling is not conceptualized as an “altruistic” action (although in some cases it can be) but as part of a strategy for *joint action optimization* [20]. The minimization of uncertainty is important in individualistic planning. Here we extend this argument to joint actions, arguing that if performers maintain a model of the perceiver agent, minimizing his uncertainty is part of the optimization and this entails some form of signaling and communication. Thus, sensorimotor communication emerges as part of a strategy that enhances coordination and the success of joint actions.

### The Importance of Signaling in Joint Actions

Recent research in social neuroscience has revealed that understanding the intentions of co-actors and predicting their next actions are fundamental for successful joint actions and social interactions, cooperative and competitive. In joint task such as building something together or running a dialogue, action understanding helps planning complementary actions and contributes to the success of the joint goal. Furthermore, predictive mechanisms help the real-time coordination of one’s own and the co-actor’s actions [36]–[41].

However, action understanding and prediction are hard and often under-constrained computational problems, and it is still unclear how humans (and other animals) solve them in real time while at the same time planning their complementary or competitive actions [42]–[46]. It has been argued that action and intention recognition are facilitated in joint action but also more in general in social set-ups because co-actors tend to automatically “align” at multiple levels, imitate each other, and share representations; in turn, this facilitates prediction, understanding, and ultimately coordination [42], [45], [47]. Dynamic theories of joint action argue that due to alignment processes and the synchronization of behavior, coordination dynamics could be self-reinforcing, so that joint actions (or at least those requiring only immediate coordination) do not require cognitive representations [48], [49]. Despite the importance of automatic mechanisms, we argue that co-actors adopt also *intentional* strategies to enhance coordination. In keeping with this view, human experiments show that signaling is versatile and takes into account the communicative implications of the executed actions, its ambiguity and its costs, as well as the uncertainty of the co-actor [17], [19], [20], and for this can be considered an intentional strategy, although not necessarily conscious.

We argue that signaling strategies help solving coordination problems and joint actions by making actions easier to predict and disambiguate. In the long run, signaling actions can also support common ground formation by disambiguating an actor’s intended *plan* rather than only her next action [3], [20], [39], [50]. Signaling actions are thus part of a joint action optimization process that takes the co-actors costs into consideration; similar examples of collaborative strategies for solving joint problems are the principle of least collaborative effort [51] and the active management of resources [52] in dialogue.

An open objective for future research is investigating empirically the multifarious methods humans use for sensorimotor communication and signaling, which are not fixed but depend on task demands and goals [53], [54]. We have focused on a specific form of signaling consisting in modifying the kinematic parameters of a given internal model; however, there are other methods to convey communicative message through one’s choice of actions, such as for example selecting a different internal model that nevertheless achieves the desired pragmatic goal [20]. Furthermore, our formulation considers the case where the alternatives are known. In certain circumstances the producer can be uncertain on the number of alternatives, or they can be too numerous to be considered all together. In these cases, the producer can decide to make the selected action easier to disambiguate by lowering its variance or by amplifying its specificities; one example is making the word “house” easier to understand by uttering “hoooouse”. Note also that although we focused principally on cooperative scenarios, signaling is widely used in competitive set-ups, too, as in the case of feinting adversaries during volleyball or soccer games [27], [28]. As for cooperative scenarios, the costs and benefits of feinting can be considered within an action optimization framework. Note that in some cases signaling actions can also be detrimental, because trajectory deviations can increase the risk of missing a target. For example, football players who want to dissimulate their penalty kick sometimes miss the target. Future studies are needed to elucidate the functioning of sensorimotor communication strategies in realistic human interactions.

### The Adaptive Value of Communication in Pragmatic Contexts

When communication is studied in purely communicative scenarios (e.g., conversations) it is tempting to argue that it maximizes information measures such as the correct reproduction of a message, as indeed assumed by prominent frameworks such as Communication Theory [55]. We studied instead communication in the context of joint actions in which the pragmatic, non-communicative goals are more prominent. In joint action set-ups the adaptive value of communication is linked to the achievement of pragmatic goals, not to information transmission per se [4], [6]–[9]. Thus, the trade-off between the costs of communication (e.g., biomechanical costs, slower performance) and its benefits for interaction success has to be considered, especially if, as in our volleyball example, the same “channels” (hand and arm movements) are used for both for action performance and communication. In our framework signaling and sensorimotor communication strategies emerge naturally from the objective of optimizing a joint goal. We believe that the proposed normative framework can contribute to shed light on the adaptive and evolutionary value of communication in terms of enhanced coordination and joint action success rather than only in terms of the maximization of information transmission.

The theory we have presented does not require that the perceiver recognizes the communicative nature of the performer’s action, i.e., that the performer intended to communicate. Still, several researchers argue that recognizing the communicative intention is be a fundamental element of pedagogical contexts [56] and linguistic communication [5]. It is worth noting that if the perceiver uses the computational framework elucidated in this article, he could be able to to distinguish a communicative from a non-communicative action by recognizing if the perceived action was generated using an “original” distribution or a “signaling” distribution. The latter is often not the optimal trajectory to achieve a pragmatic goal and this information can be used to estimate communicative intent. Future studies will need to elucidate the importance of recognizing communicative intention in joint actions, besides pedagogical contexts.

The current model has some limitations that should be addressed by future research. First, we adopted a simplified action execution model that does not consider action biomechanics, muscle activation, and detailed planning mechanisms. Furthermore, for the sake of simplicity we assumed that the costs of signaling (e.g., biomechanical costs, costs for planning unfamiliar trajectories) are proportional to the amount of the deviation from the “original” trajectory. These limitations can be overcome by introducing in our formulation more detailed action models based on optimal control theory [57], [58]. A second limitation is that the performer’s choice of the amount of signaling 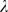 is not explicitly modeled. In the human experiment described in section 0 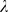 varies over time, but it remains to be empirically assessed which factors (e.g., the perceiver’s feedback, the varying biomechanical costs) affect it. A more complete approach should consider the perceiver’s behavior and the reciprocal interactions between performer and perceiver, including the fact that they can build a model of one another. Finally, the cost-benefit analysis of Eq. 4 is demanding and it is unclear if and how the brain might implement it. The more demanding part consists in calculating the perceiver’s benefits for each level of 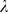. In cognitive science and neuroscience, it has been proposed that action perception can be implemented as a mental simulation that reuses the same internal model as those used for action control [59]; several computational models have been proposed that implement this process [33], [60]–[62]. A similar mechanism could allow simulating the perceiver’s action observation process. In keeping with this view,recent evidence indicates that the neural underpinnings of the ability to tailor the communicative message to the receiver could overlap with the brain substrate for intention recognition [63]–[65]. Developing computationally feasible solutions for such computations remains an important open issue.

Another promising direction for future research is linking more directly the proposed approach to established frameworks in computational motor control and social interaction. The proposed theory can be linked to existing models of Bayesian *decision theory* and reward-directed motor control [66]–[68] by reformulating the costs and benefits of signaling in terms of a reward function. In this framework, the choice of signaling or not signaling would result from the optimization of the reward function rather than eq. 4. The proposed theory would also benefit from a linkage with *game theory*, which is an established framework for studying coordination problems. in this vein, a few recent studies linked sensorimotor or cognitive processes to equivalent game-theoretic concepts, but did not consider communication [69]–[71]. Different from game theory, our framework aims at clarifying the micro-dynamics and cognitive mechanisms that support joint actions; linking game-theoretic concepts to cognitive mechanisms could help realizing theories of coordination dynamics that span several levels or explanation. Our proposed method of *joint action optimization* resonates with game-theoretic concepts of *team reasoning* [72], [73]; exploring the linkage between these concepts is an open objective for future research.
